# Auditory Spatial Perception without Vision

**DOI:** 10.3389/fpsyg.2016.01960

**Published:** 2016-12-20

**Authors:** Patrice Voss

**Affiliations:** Cognitive Neuroscience Unit, Department of Neurology and Neurosurgery, Montreal Neurological Institute – McGill UniversityMontreal, QC, Canada

**Keywords:** spatial hearing, vision disorders, blindness, auditory perception, critical period (psychology)

## Abstract

Valuable insights into the role played by visual experience in shaping spatial representations can be gained by studying the effects of visual deprivation on the remaining sensory modalities. For instance, it has long been debated how spatial hearing evolves in the absence of visual input. While several anecdotal accounts tend to associate complete blindness with exceptional hearing abilities, experimental evidence supporting such claims is, however, matched by nearly equal amounts of evidence documenting spatial hearing deficits. The purpose of this review is to summarize the key findings which support either enhancements or deficits in spatial hearing observed following visual loss and to provide a conceptual framework that isolates the specific conditions under which they occur. Available evidence will be examined in terms of spatial dimensions (horizontal, vertical, and depth perception) and in terms of frames of reference (egocentric and allocentric). Evidence suggests that while early blind individuals show superior spatial hearing in the horizontal plane, they also show significant deficits in the vertical plane. Potential explanations underlying these contrasting findings will be discussed. Early blind individuals also show spatial hearing impairments when performing tasks that require the use of an allocentric frame of reference. Results obtained with late-onset blind individuals suggest that early visual experience plays a key role in the development of both spatial hearing enhancements and deficits.

## Introduction

Our sense of vision provides us with the most detailed information about the spatial configuration of our environment. This visual dominance stems in part from the brain receiving high-resolution spatial information directly from the retina that is coded topographically throughout the visual pathway. While other modalities extract spatial information in a similar manner (e.g., tactile, vestibular, and proprioceptive modalities), they are body-centric and do not provide reliable information beyond personal and peripersonal space (i.e., beyond the reach of any limb). However, there are exceptions to this rule, such as when using sensory-substitution devices to translate visual information into tactile input that can be perceived, for instance, on either the tongue or the back (e.g., Bach-y-Rita et al., 1969; Chebat et al., 2007). The auditory system, like the visual system, also provides relevant spatial information regarding more distant regions of space. Localization information, however, is based on the detection and interpretation of auditory spatial cues that vary in their usefulness (for reviews, see Middlebrooks and Green, 1991; Schnupp et al., 2010). Consequently, vision has often been thought to be essential for many aspects of spatial cognition and perception, and it has been often suggested that the absence of visual input might constitute a significant detriment to the ability to form accurate spatial representations. Two opposing views have emerged from early experimental findings (for review, see Rauschecker, 1995). The first view provides support for a perceptual deficit hypothesis whereby in the absence of visual input, individuals may develop cognitive spatial deficits in other sensory modalities (Axelrod, 1959; Jones, 1975). This hypothesis was supported by a large body of animal work that illustrated the importance of visual feedback in auditory spatial learning (Knudsen, 1985; King et al., 1988; Withington-Wray et al., 1990; Knudsen et al., 1991; Heffner and Heffner, 1992) and for the normal development of acoustic spatial maps in the superior colliculus (Knudsen, 1988; Withington, 1992; King and Carlile, 1993).

The opposing point of view supports a *sensory compensation hypothesis whereby* blind individuals develop exceptional perceptual abilities within their remaining sensory modalities to compensate for the visual loss (Rice, 1970; Miller, 1992). Pre-existing anecdotal support comes from, among others, both Denis Diderot (1749) in his *Lettre sur les Aveugles* and William James who dedicated a full chapter to this question in his 19th-century essay “The Principles of Psychology” (James, 1890). Experimental support was also provided by several animal (Rauschecker and Korte, 1993; King and Parsons, 1999) and human (Niemeyer and Starlinger, 1981; Muchnik et al., 1991) studies that reported enhanced sound localization abilities following prolonged visual deprivation. Subsequent studies provided evidence that corroborated the compensation hypothesis by demonstrating superior spatial hearing abilities in early blind individuals (Lessard et al., 1998; Röder et al., 1999; Leclerc et al., 2000; see also Voss et al., 2010 for a review), and supported the view that blind individuals can develop heightened abilities in their remaining sensory abilities. This hypothesis further gained traction with the growing body of evidence showing that these enhanced spatial hearing abilities are subserved by crossmodal plasticity (for reviews, see Collignon et al., 2009; Voss and Zatorre, 2012). Spatial hearing tasks have been shown to elicit significant activation within the visual cortex of early blind individuals (Weeks et al., 2000; Gougoux et al., 2005; Renier et al., 2010; Collignon et al., 2011), and individual localization abilities have been shown to strongly correlate with the magnitude of visual cortex activity (Gougoux et al., 2005; Voss et al., 2008, 2011). How auditory input comes to be processed in the visual cortex of the blind remains unclear, however, there is a growing body of animal tracer (Falchier et al., 2002, 2010; Clavagnier et al., 2004) and neuroimaging evidence (Klinge et al., 2010; Collignon et al., 2011, 2013) suggesting that corticocortical pathways between auditory and visual cortices may underlie the crossmodal processing. Despite this body of evidence supporting the compensation hypothesis, a clearer picture emerges when we take a closer look at the specific conditions under which enhanced spatial hearing abilities are observed.

## Dimensions of Space

Our spatial environment can be divided into distinct dimensions. With regards to spatial hearing, it is typically divided into the horizontal, vertical, and depth planes. The most studied dimension is the horizontal plane, likely due in part to its relevance for aurally localizing objects for navigation and wayfinding purposes. In the horizontal plane, the blind have been shown to possess similar spatial hearing abilities to the sighted in the frontal hemifield (e.g., within the region approximately overlapping the visual field). In contrast, however, the blind display more accurate localization in peripheral auditory space, particularly for sound sources straddling the interaural axis (Röder et al., 1999; Voss et al., 2004; Desprès et al., 2005).

A seminal study identified a marked difference in spatial hearing ability between early blind and sighted individuals when having to localize sounds under monaural listening conditions (e.g., with one ear occluded). Lessard et al. (1998) showed that the blind are significantly better at monaurally localizing sounds coming from sources ipsilateral to the occluded ear (see also Gougoux et al., 2005). This monaural superiority, combined with more accurate localization abilities in peripheral auditory space, point toward the better utilization of a specific set of localization cues by early blind individuals. Doucet et al. (2005) and Voss et al. (2011), using distinct but complementary methodologies, showed that a higher sensitivity to spectral cues likely underpins the superior localization abilities of the early blind in the horizontal plane. Spectral cues result from the location-specific head-dependant filtering of the incoming sound by the outer ear (Shaw, 1966). The resulting spectral profile is altered by the pinna in a manner that is specific to the direction of the incoming sound wave. While it has also been shown that the blind display higher sensitivity to binaural sound location cues compared to sighted individuals (Nilsson and Schenkman, 2016), such cues are absent in monaural listening conditions and are not always reliable in peripheral auditory space (Jin et al., 1999), suggesting that these binaural cues are unlikely to underlie the spatial hearing enhancements observed on the horizontal plane in blind individuals.

The spectral cue hypothesis, however, is challenged by spatial hearing findings in the vertical plane. Although localization ability in the vertical plane is also believed to rest primarily on spectral cues (Middlebrooks and Green, 1991; Blauert, 1997), early blind individuals have been shown to be worse than sighted individuals when localizing sound targets in the vertical mid-sagittal plane (Zwiers et al., 2001; Lewald, 2002). Voss et al. (2015) recently attempted to resolve this discrepancy by comparing the ability of early blind individuals to localize sounds in both the horizontal and vertical plane. The results confirmed both sets of previous findings: on average, the blind are better at localizing sounds monaurally in the horizontal plane and display deficits when localizing in the vertical plane. The novel finding, however, was that performance in both tasks was inversely correlated for the blind: those who displayed the highest accuracy in the horizontal plane were also the ones with the largest deficit when localizing in the vertical plane. Such a correlation was not observed in sighted individuals. This finding not only argues against the idea of generalized auditory spatial perceptual enhancements in the blind, but also suggests the possibility of a trade-off in the localization proficiency between the two auditory spatial planes, such that learning to use monaural cues for localization in the horizontal plane comes at the expense of using them to localize in the vertical plane. What remains unclear, however, is why such a trade-off occurs. From an ecological perspective, the enhancements observed in the horizontal plane may result from their greater relevance for navigational and wayfinding tasks. With regards to underlying mechanisms, one potential explanation may stem from the type of spectral information that is being used for each specific plane. For instance, it has been previously argued that localization in the vertical plane relies primarily on spectral notch cues, whereas localization in the horizontal plane appears to depend on the analysis of covert spectral features. A reliable cue to estimate source elevation is provided by the center frequency of a spectrum notch, which has been shown to increase systematically from about 5 to 14 kHz with corresponding increases in elevation (Hebrank and Wright, 1974; Bloom, 1977; Butler and Belendiuk, 1977). In contrast, it has been suggested that the most reliable spectral cue for determining horizontal position comes from covert peak analysis, which requires the comparison of spectral features across several source locations (Musicant and Butler, 1984; Rogers and Butler, 1992). Furthermore, these cues have been shown to be particularly helpful for resolving source locations in peripheral auditory space (Musicant and Butler, 1984; Humanski and Butler, 1988). It is, therefore, possible that blind individuals may have learned to place greater emphasis on the analysis of covert spectral cues given their importance for establishing horizontal source position, whereas sighted individuals may have learned to pay more attention to spectral notch cues for vertical localization.

Auditory depth perception has not been as extensively studied as localization, but there are nonetheless some emerging trends (for a review, see Kolarik et al., 2016). Our ability to sense depth is essential for estimating the distance that separates us from auditory sources. When having to make relative depth judgments, early blind individuals have been shown to be more accurate that sighted individuals (Voss et al., 2004), likely due to a better use of level and direct-to-reverberant ratio (DRR) auditory cues (Kolarik et al., 2013a). In contrast, the blind have been shown to be worse when having to perform absolute distance judgments (e.g., estimate the distance that separates the observer from the source; Wanet and Veraart, 1985; Kolarik et al., 2013b). Why this discrepancy between relative and absolute judgments exists is not clear. In sighted individuals, the ability to accurately aurally perceive absolute depth is much poorer than the ability to localize sounds and to localize depth visually (Loomis et al., 1998). Furthermore, the presence of visual cues has been shown to substantially improve auditory depth estimation accuracy (Anderson and Zahorik, 2014). In the absence of vision, the ability to estimate the absolute depth of sound sources might be compromised due to the lack of visual calibration of auditory spatial representations. The ability to perform relative depth judgments, however, depends primarily on the comparison of auditory cues (level or DRR) and therefore is likely not compromised by the lack of visual feedback. Therefore, spatial inferences resulting from the processing of auditory cues might be less affected by visual loss than the development of auditory spatial maps.

Evidence from echolocation studies, however, supports the idea the developing auditory spatial maps does not require visual input (for review, see Kolarik et al., 2014). Blind individuals often make use of these cues when navigating in unfamiliar environments by either passively listening to or actively creating reflecting sounds (e.g., by tapping a cane or making clicking noises). Research has shown that blind individuals are not only more sensitive to non-generated echo cues (Dufour et al., 2005; Kolarik et al., 2013a) but have also developed superior abilities to use self-generated sounds to localize objects in the environment (Rice et al., 1965; Schenkman and Nilsson, 2010, 2011).

## Frames of Reference

An alternative perspective with which we can examine the role played by vision in spatial hearing is to examine the frame of reference that is best suited to or necessary to carry out a spatial hearing task. In spatial cognition research, a reference frame defines a means of representing the locations of entities in space. The two dominant reference frames are the allocentric and the egocentric frames of reference (for reviews, see Kosslyn, 1987; Paillard, 1991; Klatzky, 1998). Egocentric frames of reference use the body or body parts as the center of the environment, whereas allocentric frames of reference are centered on external objects or on the environment itself. Multiple reports in the spatial cognition literature have suggested that, in the absence of vision, individuals primarily rely on egocentric frames of reference to carry out spatial tasks (Millar, 1994; Cattaneo et al., 2008; Coluccia et al., 2009; Corazzini et al., 2010; Pasqualotto and Proulx, 2012).

Gori et al. (2014) were among the first to provide evidence of an allocentric deficit related to spatial hearing in the blind. Early blind individuals were shown to be severely impaired when having to perform an auditory spatial bisection task in the horizontal plane. Participants had to determine whether the spatial location of a third sound source was closer to one or the other of the first two presented sound source locations. This task requires a spatial judgment that might be more anchored to an allocentric frame of reference that depends on two external auditory landmarks. In contrast, more traditional sound localization tasks can be resolved by using an egocentric frame of reference, since subjects need no other reference point other than their own position in space. This allocentric spatial hearing deficit was subsequently confirmed by several follow-up studies in both blind adults and children (Vercillo et al., 2015, 2016), suggesting the existence of dichotomic spatial hearing abilities in the early blind that depend on the frame of reference that is best suited to carry out a spatial task. Interestingly, however, Vercillo et al. (2015) also showed that blind echolocators were not impaired when having to perform a spatial bisection task, which suggests that the development of echolocation abilities may improve auditory spatial representations or the use of allocentric frames of reference.

## Insights from Spatial Cognition Studies

Support for an allocentric deficit in the early blind is provided by spatial cognition research. The majority of studies investigating the wayfinding and spatial navigation abilities of blind individuals, in particular, have provided consistent findings (for review, see Thinus-Blanc and Gaunet, 1997). For sighted individuals, wayfinding and spatial navigation have been shown to rely on both egocentric and allocentric frames of references (Millar, 1994; Klatzky, 1998; Shelton and McNamara, 2001). It is estimated that about half the population spontaneously uses an egocentric frame of reference while the other half uses an allocentric frame of reference (Iaria et al., 2003). While the early blind tend to perform tasks requiring an egocentric frame of reference as well as sighted controls (Millar, 1994; Tinti et al., 2006; Fortin et al., 2008), they generally show difficulties when the use of an allocentric frame of reference is required (Thinus-Blanc and Gaunet, 1997; Schmidt et al., 2013). Overall, the allocentric deficit observed in spatial hearing tasks is in good agreement with the findings available in the spatial cognition literature. It should be noted, however, that a recent review does provide arguments that cast doubt over the idea of a general allocentric deficit in the blind (Schinazi et al., 2016), although these concerns apply primarily to contexts not related to spatial hearing.

## Insights from Blind Individuals with Early Visual Experience

The study of the effects of late-onset of blindness may provide additional valuable information on the mechanisms that govern the development of spatial hearing in the absence of vision. The spatial hearing of late blind individuals’ was shaped by the unique combination of visual calibration during development and prolonged blindness in adulthood. The limited available evidence suggests that the spatial hearing abilities of late-blind individuals lie somewhere in between those of early blind and sighted individuals (for a review, see Voss, 2013). Unlike the early blind, there is little evidence to suggest that late blind individuals have enhanced spatial hearing abilities relative to sighted individuals. However, the evidence from studies assessing localization abilities in the horizontal plane suggests that late blind individuals may also make better use of spectral cues to localize in peripheral space (Voss et al., 2004; Fieger et al., 2006). However, there is no evidence of enhanced monaural localization abilities in the late blind, suggesting that a different explanation likely underlies their ability to localize in peripheral space (Voss et al., 2008, 2011). Overall, it would seem that late-blind individuals do not benefit from many of the spatial hearing enhancements observed in the early blind, nor do they exhibit any perceptual deficits either (e.g., Finocchietti et al., 2015). Research into how late blind individuals localize sounds on the vertical plane and their performance on spatial bisection tasks is lacking and may provide a more complete picture of spatial hearing abilities in this population. A study by Pasqualotto et al. (2013) provided evidence that late-blind individuals employ an allocentric frame of reference when completing spatial tasks whereas early blind individuals employed an egocentric frame of reference. Deficits in using allocentric frames of reference to complete spatial tasks may not appear in late-blind individuals as they can encode spatial information through auditory channels while simultaneously benefitting from the calibration obtained via previous visual experience (Ruggiero et al., 2009; Iachini et al., 2014).

## Conclusion and Future Directions

A complex relationship exists between spatial hearing and vision. Early theories proposed that blind individuals could either develop superior spatial hearing abilities to compensate for visual loss or, in contrast, demonstrate spatial hearing deficits due to a lack of calibration from the visual system. Currently available evidence suggests that both propositions are likely true. Early blind individuals are as accurate, if not more accurate, than sighted individuals when having to localize sound sources on the horizontal plane but show deficits when localizing sound sources on the vertical plane or when estimating the absolute distance of auditory cues. In fact, recent evidence suggests a trade-off in the localization proficiency of early blind individuals between the horizontal and vertical planes (Voss et al., 2015), such that the more accurate an individual is in one plane, the worse that same individual is in the other plane. Why this trade-off occurs remains unclear, but it might stem from the greater ecological benefit of being accurate in the horizontal plane. When spatial hearing tasks require the use of an egocentric frame of reference, early blind individuals display comparable or superior perceptual abilities. However, they display significant deficits when the use of an allocentric frame of reference is required (e.g., for a spatial bisection task).

Evidence obtained with late-blind individuals suggests that while the presence of visual input early in life prevents the development of spatial hearing deficits, it also limits the emergence of perceptual enhancements. Several aspects of spatial hearing, however, remain to be investigated in the late blind. In particular, data is lacking concerning their ability to localize sounds in the vertical plane and to perform auditory spatial bisection tasks. Predictions can still nonetheless be made based on available evidence. For instance, a trade-off in localization proficiency between the horizontal and vertical planes would probably not be observed given that late-blind individuals do not exhibit better monaural localization abilities (Voss et al., 2006, 2008, 2011). Similarly, based on the lack of evidence supporting allocentric deficits in the late blind (Pasqualotto and Proulx, 2012), they should not show deficits when performing auditory spatial bisection tasks.

Despite the evidence accumulated in recent years, it remains unclear to what extent the described perceptual consequences of early blindness translate to real-world settings. Most of the presented findings have been observed under experimental conditions with limited ecological validity. To properly ascertain the real-world abilities of individuals with complete blindness, there is a need to evaluate more ecologically relevant and useful metrics. The ability to track dynamic sounds in space, for instance, and the ability to localize sounds in noisy environments appear to be important elements to investigate. While a few studies have started to investigate these metrics (Lewald, 2013; Finocchietti et al., 2015), it remains difficult, however, to draw any reliable conclusions given the limited amount of data available. Similarly, most spatial hearing experiments have been performed in anechoic environments, which are rarely found outside of the laboratory. As highlighted earlier, the fact that blind individuals are more sensitive to echoic cues (Dufour et al., 2005) means that their performance on spatial hearing tasks in an echoic environment maybe enhanced compared to an anechoic environment. Although there is evidence that blind individuals can better extract speech information from noise more efficiently than sighted controls (Rokem and Ahissar, 2009), their ability to localize sounds in the presence of background noise has not been thoroughly investigated.

Blindfolding sighted subjects might put them at a disadvantage compared to blind individuals and also might artificially inflate the difference in performance between them. Tabry et al. (2013) showed that blindfolding reduces localization accuracy in sighted individuals, and this was particularly true for the horizontal plane (compared to the vertical plane) and when having to localize sounds via head pointing (compared to hand pointing). As such, great care should be taken when designing experimental procedures for assessing certain spatial dimensions to reduce the impact of methodologically induced biases on the results.

Finally, the finding that short-term visual deprivation (as little as a few hours) in sighted individuals can improve auditory localization (Lewald, 2007) is at odds with the lack of documented improvements observed in late-blind individuals (Voss, 2013). While the spatial hearing benefit of transient visual deprivation is consistent with data investigating other sensory abilities (Facchini and Aglioti, 2003; Landry et al., 2013; Pagé et al., 2016), it is currently the only study having looked at spatial hearing. Further studies are required to ascertain the underlying processes involved in spatial hearing enhancements caused by visual loss and to further our understanding of the effects of transient sensory deprivation.

## Authors Contributions

The author confirms being the sole contributor of this work and approved it for publication.

## Conflict of Interest Statement

The author declares that the research was conducted in the absence of any commercial or financial relationships that could be construed as a potential conflict of interest.
